# Coordinating the Provision of Health Services in Humanitarian Crises: a Systematic Review of Suggested Models

**DOI:** 10.1371/currents.dis.95e78d5a93bbf99fca68be64826575fa

**Published:** 2016-08-03

**Authors:** Tamara Lotfi, Lama Bou-Karroum, Andrea Darzi, Rayan Hajjar, Ahmed El Rahyel, Jamale El Eid, Mira Itani, Hneine Brax, Chaza Akik, Mona Osman, Ghayda Hassan, Fadi El-Jardali, Elie Akl

**Affiliations:** Clinical Research Institute, American University of Beirut, Beirut, Lebanon; Health Management and Policy, American University of Beirut, Beirut, Lebanon; AUB GRADE Center, Clinical Research Institute, American University of Beirut, Beirut, Lebanon; American University of Beirut, Beirut, Lebanon; American University of Beirut, Beirut, Lebanon; Human Research Protection Program, American University of Beirut, Beirut, Lebanon; Biology, Faculty of Arts and Sciences, American University of Beirut, Beirut, Lebanon; Medicine Faculty, Saint Joseph University, Beirut, Lebanon; Center for Research on Population and Health, American University of Beirut, Beirut, Lebanon; Department of Family Medicine, American University of Beirut, Beirut, Lebanon; Psychology, Uqam, Montreal, Quebec, Canada; Health Management and Policy, American University of Beirut, Beirut, Lebanon; Department of Internal Medicine, Faculty of Medicine, American University of Beirut, Lebanon

## Abstract

Background: Our objective was to identify published models of coordination between entities funding or delivering health services in humanitarian crises, whether the coordination took place during or after the crises.

Methods: We included reports describing models of coordination in sufficient detail to allow reproducibility. We also included reports describing implementation of identified models, as case studies. We searched Medline, PubMed, EMBASE, Cochrane Central Register of Controlled Trials, CINAHL, PsycINFO, and the WHO Global Health Library. We also searched websites of relevant organizations. We followed standard systematic review methodology.

Results: Our search captured 14,309 citations. The screening process identified 34 eligible papers describing five models of coordination of delivering health services: the “Cluster Approach” (with 16 case studies), the 4Ws “Who is Where, When, doing What” mapping tool (with four case studies), the “Sphere Project” (with two case studies), the “5x5” model (with one case study), and the “model of information coordination” (with one case study). The 4Ws and the 5x5 focus on coordination of services for mental health, the remaining models do not focus on a specific health topic. The Cluster approach appears to be the most widely used. One case study was a mixed implementation of the Cluster approach and the Sphere model. We identified no model of coordination for funding of health service.

Conclusion: This systematic review identified five proposed coordination models that have been implemented by entities funding or delivering health service in humanitarian crises. There is a need to compare the effect of these different models on outcomes such as availability of and access to health services.

## Background

National and international humanitarian relief organizations play a significant role in humanitarian crises.^1^^,^ 2 For example, after the Asian Tsunami in 2004, relief operations involved the government institutions of the concerned countries, Non-Government Organizations (NGOs), United Nations (UN) agencies, and disaster relief teams from aiding nations^3^ with an estimated total number of over a 1000 actors.^4^ Coordination between these organizations in planning and adapting policies is essential to cope with these crises and enhance the ability to deal with them.^5^

There is evidence of lack of coordination between organizations providing health services in public health emergencies.^4^^,^ 6 For example, the lack of coordination between stakeholders complicated food delivery in the humanitarian crises in Iraq, Darfur and Palestine.^7^ Another example is the response to the Haiti earthquake, which was described as worst natural disaster in modern history, that lacked coordination and resources.^8^

Studies have reported the importance of providing adequate support to the relevant national authorities at the beginning of a humanitarian crisis in order to optimize the use of available resources to establish a coordination mechanism.^7^ Allowing stakeholders to work collectively creates an enabling environment for coordinating both information and action, facilitating the implementation of effective interventions and the provision of equitable assistance to those in need.^7^^,^ 9 ^,^ 10

Major organizations, such as the UN, Red Cross and governmental and non-governmental agencies have attempted to form mechanisms and frameworks of coordination.^9^ It would be important for a group of organizations contemplating a coordination mechanism, to be aware of and understand the different coordination models.

Our objective was to identify published models of coordination between entities funding or delivering health services in humanitarian crises, whether the coordination took place during or after the crises.

## Methods

**Eligibility criteria**

We included a study irrespective of its design, as long as it provided a detailed description of a coordination process or model. The types of organizations of interest included UN agencies, local and international organizations and agencies including NGOs, governmental agencies and bodies. We included different settings such as humanitarian crises, whether the coordination took place during or after the crises. We did not exclude studies that were not published in English.

**Search strategy**

We registered a protocol for this review in PROSPERO International prospective register of systematic reviews under number PROSPERO2014:CRD42014009267. We used the electronic databases (Medline, PubMed, EMBASE, Cochrane Central Register of Controlled Trials, CINAHL, PsycINFO, and the WHO Global Health Library) and websites of relevant organizations to run our search strategy (Appendix 1). We did not use any language restrictions.

**Selection of studies**

We followed the standard systematic review methodology: we screened the titles and abstracts of identified citations for potential eligibility in duplicate and independently. We retrieved the full texts of citations considered as potentially eligible by at least one of the two reviewers. Then, we screened the full texts in duplicate and independently for eligibility. We resolved disagreements by discussion or with the help of a third reviewer. We used a standardized and pilot tested screening form and completed calibration exercises.

**Data collection**

We abstracted data independently and in duplicate using standardized and pilot tested data abstraction form. We resolved disagreements by discussion or with the help of a third reviewer. We abstracted the following data: the study ID, the name of the model, the setting it was implemented in, and the description of the model.

**Data synthesis**

Given the qualitative nature of the data, we synthesized and reported the findings narratively. First, we narratively described both the full and partial coordination models. Full coordination models refer to models that attempted to cover all aspects of interaction between corresponding agencies and bodies while partial models covered only one aspect of interaction such as information coordination. In addition we presented in a tabular format the case studies relevant to each of these models.

## Results

**Selection of studies**

Figure 1 shows the study flow. Of the 14,309 citations identified by the electronic literature search, 34 papers met our inclusion criteria and described five models of coordination of delivering health services: the Cluster approach^8^^,^ 9 ^,^ 11 ^-^ 32 ; the 4Ws “Who is Where, When, doing What” mapping tool 33 ^-^ 36; the Sphere project 9 ^,^ 23 ^,^ 37 ^,^ 38; the 5x5 model 39; and a model of information coordination^40^. We did not identify any model of coordination for funding health services. We excluded 412 papers for the following reasons: not dealing with a model of coordination (n=311); not describing how coordination was done (n= 4); conference abstracts (n= 99).

**Study Flow Diagram:**
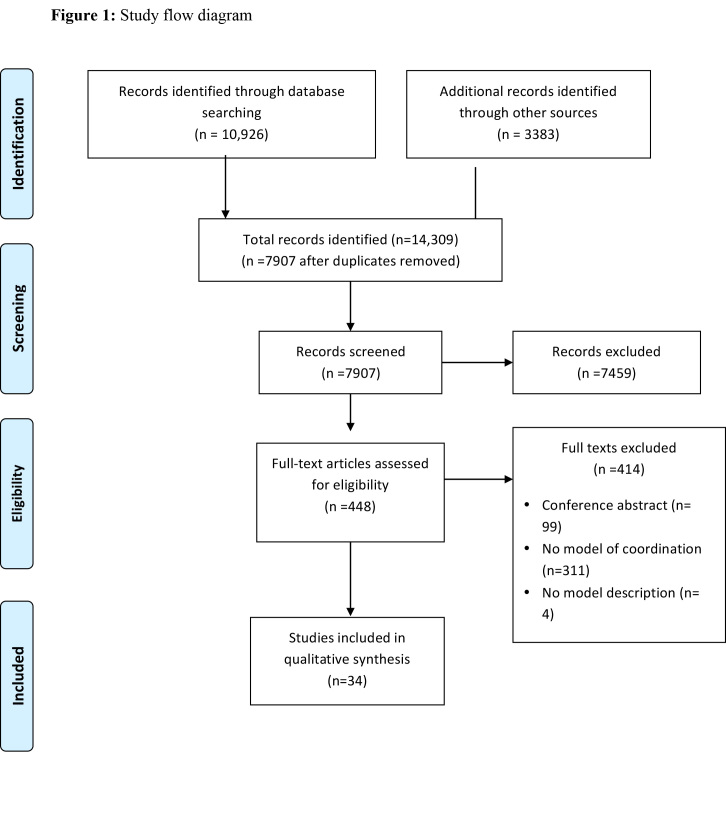

We describe below the five models in full details. Table 1 compares the main characteristics of the five models. Appendix 2 presents the identified case studies for the different models: the Cluster approach (n=16); 4Ws (n=4); the Sphere project (n=2); the 5x5 model (n=1) and the model of information coordination (n=1).

**Figure:**
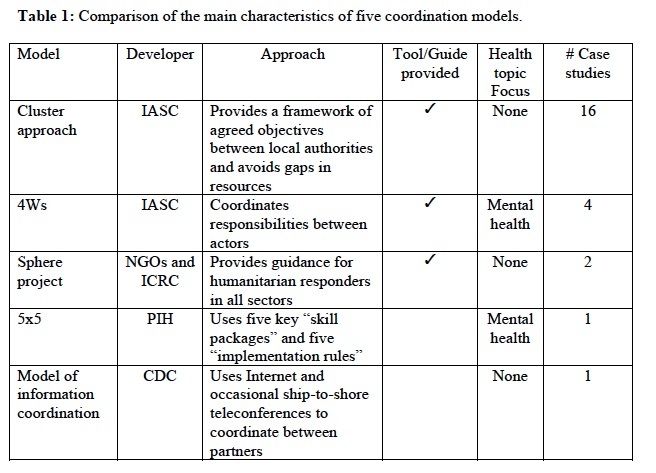
**Table 1. Comparison of the main characteristics of five coordination models.** (IASC: Inter-Agency Standing Committee; NGOs: Non-Governmental Organizations; ICRC: International Red Cross and Red Crescent; PIH: Partners in Health; CDC: Center for Disease Control and Prevention)

**The cluster approach**

In 1992, key UN and non-UN humanitarian partners, established the Inter-Agency Standing Committee (IASC) as the “primary mechanism for inter-agency coordination of humanitarian assistance”. In 2005, the IASC worked with the UN Emergency Relief Coordinator to develop the cluster approach as a “way of organizing coordination and cooperation among humanitarian actors to facilitate joint strategic planning”.^11^^-^ 13 ^,^ 31 ^,^ 32 The Cluster aims to support and match the efforts of national authorities in critical areas of preparedness and response within a framework of agreed objectives. It also aims to avoid gaps and/or overlap in the resources and international humanitarian response.

Figure 2 shows how the Cluster system works. A Cluster forms at the country level under the overall leadership of the humanitarian coordinator, and includes multiple national and international agencies working together within a specific sector of emergency response. A Cluster Lead Agency (CLA) is assigned for each sector: United Nations Children’s Fund (UNICEF) for nutrition and for water and sanitation, the World Health Organization (WHO) for health, and the United Nations High Commissioner for Refugees (UNHCR) and the International Federation for Red Cross and Red Crescent (IFRC) for emergency shelter. Moreover, the lead agency or co-lead agencies are held accountable, through this approach, for the performance of their cluster by being responsible to ensure adequate coordination of activities by partners involved in its specified area.^29^ Figure 3 shows the inter-organizational functioning of a Cluster approach with further narrative details provided in Appendix 3.

**Diagram illustrating how the Cluster system works:**
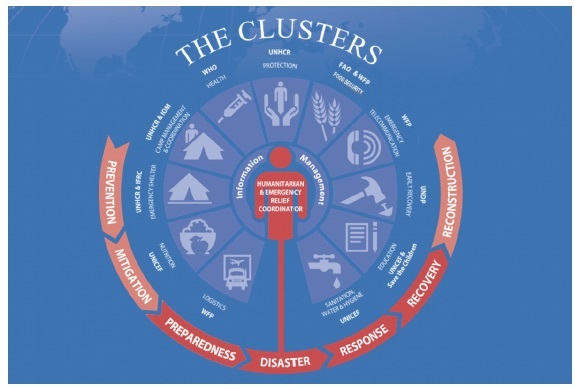
Photo credit: Office for the Coordination of Humanitarian Affairs^49^

**Inter-organizational functioning of a Cluster approach Humanitarian health response coordination framework in Pakistan:**
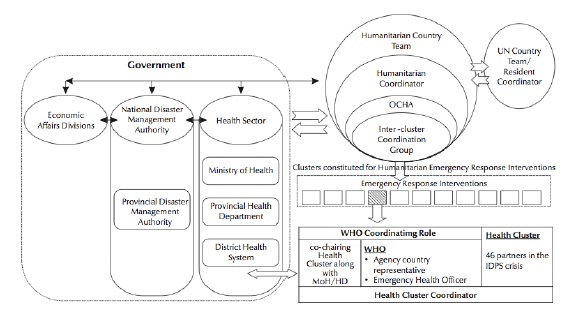
(IDPs = Internally Displaced Persons; OCHA = UN Office for Coordinating Humanitarian Affairs)

Appendix 2 describes 16 papers reporting on 19 case studies where the Cluster approach was implemented.

**The 4Ws**

In 2007, the IASC developed through its Reference Group on Mental Health and Psychosocial Support (MHPSS) the “Who is Where, When, doing What” (4Ws) tool.^33^ The IASC developed this tool to help in the coordination of responsibilities between the MHPSS actors responding to the Iraqi refugees crisis in Jordan following the 2003 Iraqi war.

The 4Ws tool included a one-page introduction and three activity spreadsheets: one for information about the organization, one for details of activities, and the last one for 11 pre-defined MHPSS activities and corresponding sub-activities.^34^^,^ 36 ^,^ 41 Three mapping exercises were conducted, and aimed to: 1) Map MHPSS activities in Jordan; 2) Recommend changes to the tool, based on field experience; 3) Present the findings of the mapping to the Jordan MHPSS Coordination Group.^36^ After each mapping exercise, adjustments were made to the activity spreadsheet.^36^

As an illustrative example, and in the case of Jordan, the tool identified the following:^36^

- Who, the participating actors: number and names of organizations contacted and those that participated;
- Where, the geographic locations: where the highest and lowest concentration of activities were located;
- When, the initiation and duration of activities: the tool tracked 30 day cycles of activities in 2009;
- What, the types of MHPSS activities: 11 activities (e.g., information dissemination to the community at large; safe spaces; facilitating conditions for community mobilization, community organization, community ownership or community control over emergency relief in general).

The IASC provides a guide on when and how to apply this tool (Appendix 4).^33^

**The Sphere Project**

In 1997, a group of NGOs and the International Red Cross and Red Crescent (ICRC) Movement developed the Sphere Project following the 1994 Rwandan genocide. The project aim was to provide guidance for humanitarian responders in all sectors.^27^ It is considered the “first collaborative initiative to produce globally applicable minimum standards for humanitarian response”. Sphere encourages providing a coordination framework between governmental organizations for international and local disaster relief. 22 ^,^ 38 ^,^ 42

The Sphere project developed a tool for “field-based inter-agency coordination” which consisted of: 1) binding principles of engagement; 2) protocol for assumption of responsibilities; 3) health-sector gap identification and 4) health-sector components summaries. The project also identified a set of “minimum standards in health action” for evidence-based and sector-wide consensus on best practices in humanitarian response. Appendix 5 lists the key actions in leadership and coordination, part of those minimum standards.

**5x5 model**

In the aftermath of the Haiti earthquake, Haitian and American nationals working for Partners in Health (PIH) developed the “5x5” intervention model to manage mental health services delivered by their organization.^39^ The name of the intervention refers to five key “skill packages” and five “implementation rules”.

The five key “skill packages” aim to provide mental health-specific platform to apply algorithms for common disorders. These packages are consistent with the WHO mental health intervention guide in non-specialist health settings and include: 1) case finding, engagement, follow-up, and psycho-education; 2) targeted psychological interventions; 3) medication management; 4) supervision and consultation; 5) quality oversight.

The five “implementation rules” consist of the following: 1) assess context first; 2) identify priority care pathways; 3) specify decision-support tools, supervision, and triage rules; 4) use quality-improvement practices; 5) plan for sustainability and capacity building; and thus the name 5x5 model.

**A model of information coordination**

In 2004, and in response to the Tsunami of the shores of the Indian Ocean, the Center for Disease Control and Prevention (CDC) formed the Responder Resilience and Mental Health Team to contribute to the humanitarian relief efforts.^40^ Because of the complex relief efforts, there was a need for coordination between partners through the Internet and occasional ship-to-shore teleconferences. It was important to inform the emergency operations command about the psychosocial relief efforts through reports, critical resource documents on traumatic exposure metrics and intervention manuals with intervention mapping strategies.

**Coordination for funding health services**

Although we did not identify any model of coordination for funding health services, we identified one paper discussing coordinating funds in humanitarian crises. This paper presented the results of a conference held by WHO in Phuket, Thailand, May 2005 to assess the Health Aspects of the Tsunami Disaster in Asia, December 2004. One problem relied in managing the outpouring resources of aid. Four recommendations were brought up in this conference: 1) to create a financial monitoring system reliable and cost-effective for all stakeholders; 2) to require targeted funding and address constraints of pooled funding; 3) to compare the Tsunami funding to principles of best practices and evaluate it; 4) to better coordination within and across organizations and between donors.^43^

## Discussion

Our systematic review aimed to identify models of coordination between entities funding or delivering health services in humanitarian crises.

We found five models of coordination of delivering health services and no model of coordination for funding these services. While the 4Ws and the 5x5 focus on coordination of services for mental health, the remaining models do not focus on a specific health topic. The Cluster approach appears to be the most widely used, at least based on the number of identified case studies using it (n=16 out of 24). The Sphere Project was used along with the cluster approach in one case study.

There might be different explanations for why the Cluster approach was the most widely reported to be used. First, it is the first approach to have been developed and reported. Second, the developer of the approach being the UN system has probably given it more visibility than other approaches. In addition, there is some evidence that this approach may improve coordination among organizations, particularly those working on sexual and reproductive health.^47^

In fact, our group has produced one systematic review assessing the effectiveness of models of coordination.^44^ The review identified only four evaluation studies providing very low quality evidence,^45^^-^ 48 and only one of these assessed a formally defined coordination model, which was the cluster approach.^47^ Those studies found that information coordination may be effective in improving health systems inputs; and that management coordination (e.g., with the cluster model) may improve health system inputs and access.^44^

Our study has a number of strengths. First, it is the first study describing models for coordinating health services in humanitarian crises. Second, we followed a systematic review methodology, including a search of non-peer reviewed literature. One limitation of the study is the exclusion of non-English published papers. However, it is unlikely that an eligible study was published in a language different than English given the usual involvement of international organization in these situations.

While the authors of the models call for using them across emergency settings, the case studies suggest a potential pattern for using certain models for specific types of emergencies. For example, the Cluster and 4Ws approaches have been used in emergencies related to both war and natural disasters while the rest of the models have been reportedly used only in emergencies related to natural disasters.

Irrespective of the specific coordination model, their use in general might be challenging during or in the aftermath of humanitarian crises for a number of reasons. The nature of the crisis itself and the urgency to deliver services might not allow organizations to dedicate the needed time or resources to coordinate their efforts. Also, these organizations might not be aware of such models, or might not have the needed expertise to implement them. Finally, the cultures of the individual organizations might conflict and negatively affect any will or effort to coordinate.

In conclusion, five coordination models have been implemented worldwide in different disasters in order to coordinate the delivery of health services. These results should serve policymakers, and administrators of entities delivering health services during and post-humanitarian crises to choose from a number of options on how to coordinate their efforts. It is challenging to provide specific guidance on which model to use. However, we suggest that decision makers prioritize models that, based on the presented case studies, have been used in settings similar to theirs, such as the type of disaster or the countries in which the crisis is taking place.

There is a need to conduct further research to assess the effectiveness and efficiency of the identified models, specifically in terms of availability of healthcare services and access to health services.

## Appendices

**Appendix 1:** **Medline, EMBASE, Scopus, CINAHL, WHO Global Health Library, PsychInfo, and Cochrane Search strategies**

***Medline***

Database: Ovid MEDLINE(R) <1946 to March Week 1 2014>

Search Strategy:

1 exp Refugees/ (6558)

2 refugee*.ti,ab. (5394)

3 exp War/ (30340)

4 (war or wars).ti,ab. (25791)

5 disasters/ or exp disaster planning/ or exp emergency shelter/ or exp mass casualty incidents/ (23240)

6 (disaster* or tsunami* or earthquake* or volcan* or hurricane* or cyclone*).ti,ab. (20082)

7 (mass adj2 casualt*).ti,ab. (1355)

8 exp earthquakes/ or exp tsunamis/ or exp volcanic eruptions/ (2728)

9 ((conflict* adj3 (area* or zone* or setting* or region)) or (conflict-affected adj3 (area* or zone*or setting* or region)) or armed-conflict* or (armed adj3 conflict*) or (conflict adj3 ethnic) or post-conflict or postconflict or (military adj3 conflict) or "post conflict").ti,ab. (1770)

10 ((internal* adj2 (displace* or dis-place*)) or (forcibl* adj2 (displace* or dis-place*))).ti,ab. (422)

11 or/1-10 (84689)

12 (coordinat* or co-ordinat* or cooperat* or co-operat* or collaborat*).ti,ab. (297694)

13 exp cooperative behavior/ (28026)

14 international cooperation/ or medical missions, official/ (39498)

15 or/12-14 (346524)

16 organizations/ or exp charities/ or government/ or exp "united states dept. of health and human services"/ or exp local government/ or exp state government/ or exp government agencies/ or exp international agencies/ or organizations, nonprofit/ or foundations/ or voluntary health agencies/ (155048)

17 ((international or government* or non-government* or nongovernment* or nonprofit or non-profit or donor*) adj2 (organization* or organisation* or agenc* or bodies or foundation*)).ti,ab. (15316)

18 (united adj nation*).ti,ab. (3232)

19 (red adj cross).ti,ab. (2727)

20 ("world health" adj (organization or organisation)).ti,ab. (26650)

21 exp relief work/ or exp rescue work/ (4857)

22 ((relief or rescue) adj (work or effort*)).ti,ab. (395)

23 ((foreign or humanitarian) adj2 (aid or aids)).ti,ab. (497)

24 or/16-23 (193928)

25 15 and 24 (18828)

26 ((health adj3 (cluster* or inter-cluster or zone*)) or (cluster adj2 approach)).ti,ab. (952)

27 25 or 26 (19765)

28 11 and 27 (2006)

***EMBASE***

Database: EMBASE <1980 to 2014 Week 10>

Search Strategy:

1 exp refugee/ (7321)

2 refugee*.ti,ab. (6074)

3 disaster/ or mass disaster/ or natural disaster/ or disaster planning/ (26347)

4 (disaster* or tsunami* or earthquake* or volcan* or hurricane* or cyclone*).ti,ab. (27440)

5 (mass adj2 casualt*).ti,ab. (1621)

6 exp earthquake/ (5038)

7 exp tsunami/ (1424)

8 exp volcano/ (2135)

9 war/ (24617)

10 (war or wars).ti,ab. (29491)

11 ((internal* adj2 (displace* or dis-place*)) or (forcibl* adj2 (displace* or dis-place*))).ti,ab. (548)

12 ((conflict* adj3 (area* or zone* or setting* or region)) or (conflict-affected adj3 (area* or zone*or setting* or region)) or armed-conflict* or (armed adj3 conflict*) or (conflict adj3 ethnic) or post-conflict or postconflict or (military adj3 conflict) or "post conflict").ti,ab. (2216)

13 or/1-12 (92212)

14 (coordinat* or co-ordinat* or cooperat* or co-operat* or collaborat*).ti,ab. (376241)

15 exp cooperation/ (39554)

16 coordination/ (3306)

17 exp international cooperation/ (157714)

18 or/14-17 (550057)

19 exp non profit organization/ (21069)

20 ((international or government* or non-government* or nongovernment* or nonprofit or non-profit or donor*) adj2 (organization* or organisation* or agenc* or bodies or foundation*)).ti,ab. (20577)

21 (united adj nation*).ti,ab. (4259)

22 (red adj cross).ti,ab. (3471)

23 ("world health" adj (organization or organisation)).ti,ab. (33513)

24 red cross/ (2731)

25 United Nations/ (6687)

26 world health organization/ (66809)

27 rescue work/ (573)

28 relief work/ (859)

29 ((relief or rescue) adj (work or effort*)).ti,ab. (466)

30 ((foreign or humanitarian) adj2 (aid or aids)).ti,ab. (454)

31 or/19-30 (136509)

32 18 and 31 (28322)

33 ((health adj3 (cluster* or inter-cluster or zone*)) or (cluster adj2 approach)).ti,ab. (1271)

34 32 or 33 (29576)

35 13 and 34 (2523)

***Scopus***

Search Strategy:

((TITLE-ABS-KEY(refugee* OR disaster* OR earthquake* OR tsunami* OR hurricane* OR cyclone*or war OR wars OR conflict OR conflicts OR post-conflict)) OR (TITLE-ABS-KEY(internal* W/2 displace*)) OR (TITLE-ABS-KEY(forcibl* W/2 displace*)) OR (TITLE-ABS-KEY(mass W/2 casualt*))) AND (((TITLE-ABS-KEY(coordinat* OR co-ordinat* OR cooperat* OR co-operate* OR collaborat*)) AND (((TITLE-ABS-KEY((international OR government* OR non-government* OR nongovernment* OR nonprofit OR non-profit OR donor*) W/2 (organization* OR organisation* OR agenc* OR bodies OR foundation*))) OR (TITLE-ABS-KEY(united W/0 nation*)) OR (TITLE-ABS-KEY(red W/0 cross)) OR (TITLE-ABS-KEY("world health organization" OR "world health organisation"))) OR (TITLE-ABS-KEY(foreign W/1 aid)) OR (TITLE-ABS-KEY(humanitarian W/1 aid)) OR (TITLE-ABS-KEY(rescue W/1 (work OR effort*))) OR (TITLE-ABS-KEY(relief W/1 (work OR effort*))))) OR (TITLE-ABS-KEY(health W/2 cluster)))

***CINAHL***

Search strategy:

S12 S1 AND S11

S11 S7 OR S8 OR S9

S10 ((health N2 (cluster* or inter-cluster or zone*)) or (cluster N1 approach)) AND (S8 OR S9)

S9 (health N2 (cluster* or inter-cluster or zone*)) or (cluster N1 approach)

S8 international N2 cooperation

S7 S2 AND S6

S6 (S3 OR S4 OR S5)

S5 (rescue N2 work) or (relief N2 (work or effort*)) or (foreign N2 (aid or aids)) or (humanitarian N2 (aid or aids))

S4 "united nation*" or "red cross" or (world health N1 (organization or organization))

S3 (international or government* or non-government* or nongovernment* or nonprofit or non-profit or donor*) N2 (organization* or organisation* or agenc* or bodies or foundation*)

S2 coordinat* or co-ordinat* or cooperat* or co-operat* or collaborat*

S1 refugee* or (internal* N2 displace*) or (forcibl* N2 displace*) or war or wars or conflict or conflicts or disaster* or (mass N2 casualt*) or tsunami* or earthquake* or volcan* or hurricane* or cyclone*

***WHO Global Health Library, PsycInfo and Cochrane***

Search strategy:

(refugee* or disaster* or war or wars or conflict or conflicts or post-conflict or internal* displace* or forcibl* displace* or mass casualt* or earthquake* or tsunami* or volcan*) AND ((coordinat* or co-ordinat* or cooperat* or co-operat* or collaborat*) and (foreign aid or humanitarian aid or rescue work or relief work or relief effort or ((international or government* or non-government* or nongovernment* or nonprofit or non-profit or donor*) AND (organization* or organisation* or agenc* or bodies or foundation*))))

**Appendix 2: Case studies by model**

**Figure:**
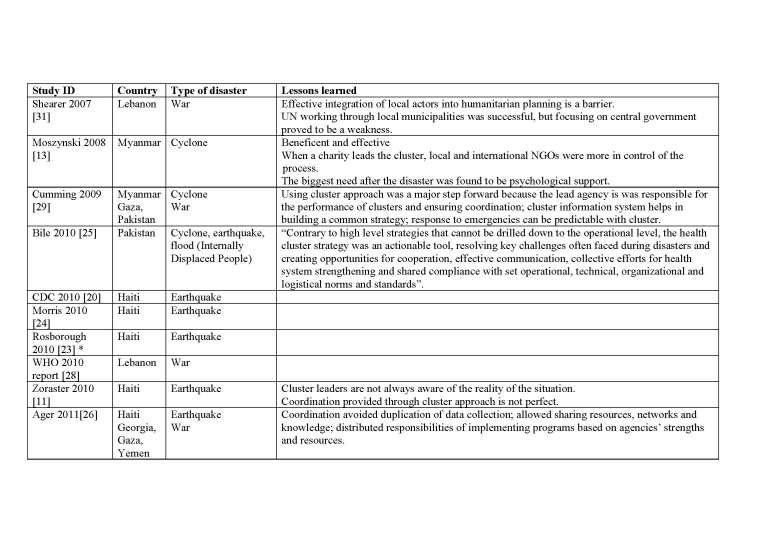
**The Cluster Approach** (* Used both the Cluster approach and the Sphere approach)

**Figure:**
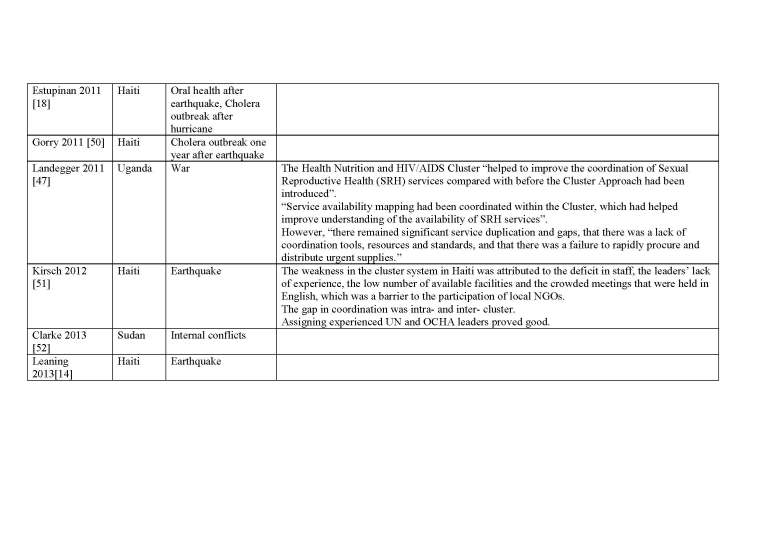
**The Cluster Approach (Continued)** (* Used both the Cluster approach and the Sphere approach)

**Figure:**
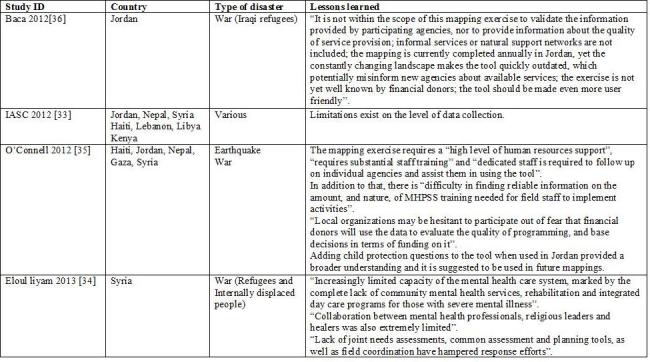
**The 4Ws**

**Figure:**
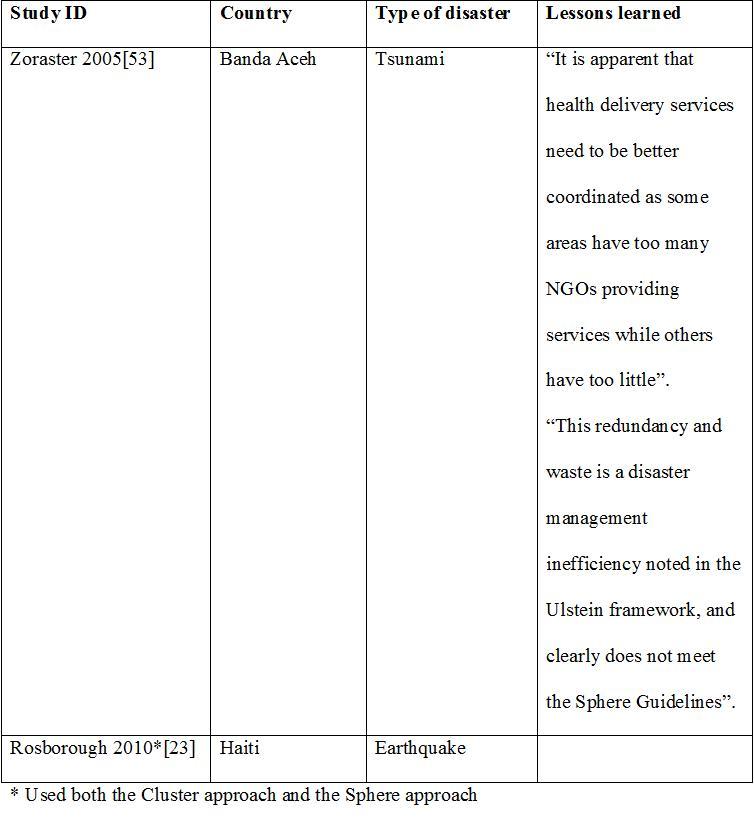
**The Sphere Project**

**Figure:**
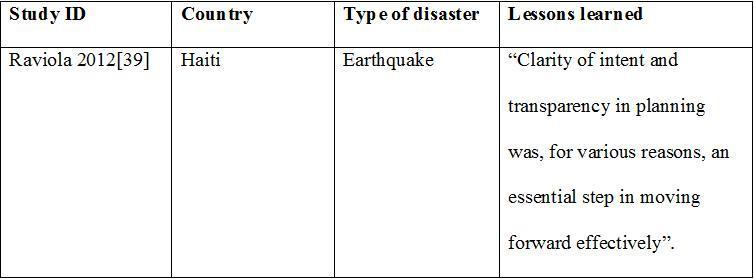
**The 5x5 Model**

**Figure:**
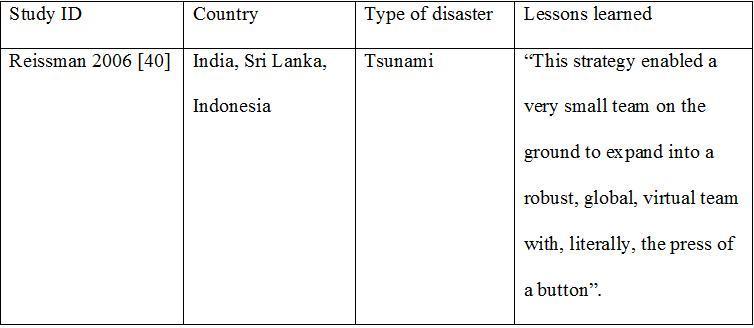
**The Information coordination Model**

**Appendix 3: Functioning of a Cluster Lead Agency**

A country-level Health Cluster includes the organizations providing health services in affected areas and the main health-sector stakeholders. In order to build on local capacities and help develop and sustain relations with relevant government and organizations involved in health-related activities, a Health Cluster lead agency bridges between the national and international actors. Ministry of health leads the coordination process in settings where it is in a strong position. In settings where the capacity of the Ministry of health to lead is compromised (such as in ongoing conflict areas), practical arrangements are made, and most of the times a ministry representative and the Cluster Lead Agency (CLA) co-chair meetings. The United Nations Office for the Coordination of Humanitarian Affairs (OCHA) is in charge of coordinating the response of the UN system to major emergencies . OCHA works closely with global lead agencies and NGOs in developing their policies and coordinating inter-cluster issues and ensures efficient system functioning on the field.

**Appendix 4: Tool used for the application of “Who is Where, When, doing What”**

**Figure:**
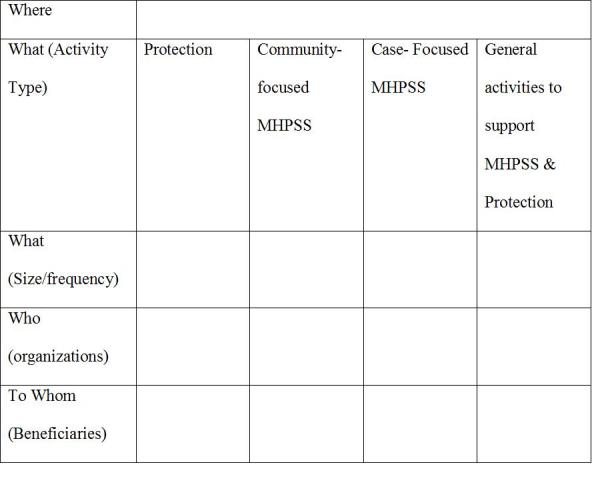
**Tool used for the application of “Who is Where, When, doing What”**

**Appendix 5: The Sphere's key actions in leadership and coordination, part of the 'minimum standards in health action'**

- Ensure that representatives of the Ministry of Health (MOH) lead or at the very least are closely involved in the health sector coordination, whenever possible;

- When the MOH lacks the necessary capacity or willingness to provide leadership in the response, an alternate agency with the requisite capacity must be identified to take the lead in health sector coordination;

- Hold regular health coordination meetings for local and external partners at central, sub-national and field levels within the health sector, and between health and other sectors and appropriate cross-cutting theme groups;

- Clarify and document the specific responsibilities and capacities of each health agency to ensure optimal coverage of the population;

-Establish working groups within the health coordination mechanism whenever a particular situation may require it (e.g. outbreak preparedness and response, reproductive health);

- Regularly produce and disseminate updates and health sector bulletins.
