# Correction: Coordinating the Provision of Health Services in Humanitarian Crises: a Systematic Review of Suggested Models

**DOI:** 10.1371/currents.dis.27a0bdc7781f35c115203b919df583eb

**Published:** 2017-02-10

**Authors:** PLOS Currents

## Correction

The thirteenth author's name is spelled incorrectly. The correct name is: Elie A Akl
